# Transection method for shortening the rat spine and spinal cord

**DOI:** 10.3892/etm.2012.841

**Published:** 2012-11-29

**Authors:** YUICHIRO YOSHIDA, HIDEO KATAOKA, TSUKASA KANCHIKU, HIDENORI SUZUKI, YASUAKI IMAJYO, HIDETOYO KATO, TOSHIHIKO TAGUCHI

**Affiliations:** 1 Department of Orthopaedic Surgery, Yamaguchi University Graduate School of Medicine; Ube, Yamaguchi, Japan; 2 Department of Orthopaedic Surgery, Yamaguchi Laborers Hospital, Ube, Yamaguchi, Japan

**Keywords:** spinal cord injury, animal model, rat

## Abstract

Previous studies have presented evidence which indicates that the regeneration of axons in the spinal cord occurs following spinal cord transection in young rats. However, in a transection-regeneration model, the completeness of the transection is often a matter of dispute. We established a method for shortening the rat spine and spinal cord to provide a spinal cord injury (SCI) model in which there was no doubt about whether the axonal transection was complete. In the future, this model may be applied to the chronic period of complete paralysis following SCI. Adult, female Wistar rats (220–250g) were used in the study. The spinal cord was exposed and a 4-mm-long segment of the spinal cord was removed at Th8. Subsequently, the Th7/8 and Th8/9 discs were cut between the stumps of the spinal cord to remove the Th8 vertebra. The stitches which had been passed through the 7th and 9th ribs bilaterally were tied gradually to bring together the stumps of the spinal cord. Almost all the rats survived until the end of the experiment. Uncoordinated movements of the hind limbs in locomotion were observed at 4 weeks after surgery. However coordinated movements of the hind limbs in locomotion were not observed until the end of the experiment. After 12 weeks, an intracardiac perfusion was performed to remove the thoracic spine and the spinal cord. There were no signs of infection. The bone fusion of the Th7 and Th9 vertebrae was observed to be complete in all specimens and the alignment of the thoracic spine was maintained. The spinal canal was also correctly reconstituted. The stumps of the spinal cord were connected. Light microscopy of the cord showed that scar tissue intervened at the connection site. Cavitation inhibiting the axonal regeneration was also observed. This model was also made on the assumption that glial scar tissue inhibits axonal regeneration in chronic SCI. Axonal regeneration was not observed across the transected spinal cord in this model. Attempts should be made to minimize the damage to the spinal cord and the surgery time for successful axonal regeneration to occur. The model developed in this study may be useful in the study of axonal regeneration in SCI.

## Introduction

Spinal cord injury (SCI) in experimental animals, particularly in adult mammalian models, is often associated with varying degrees of spontaneous functional recovery. The ability of the central nervous system to recover from injury is sometimes remarkable and at other times frustratingly limited but in either case it remains poorly understood (1–4).

Although recent studies have demonstrated axonal regeneration across the transected spinal cord, it has been suggested that the regenerated axons may not have been transected completely. This could not be resolved by the normal transected spinal cord model. We therefore established a method for shortening the rat spine and spinal cord at the thoracic level to provide a transected SCI model in which there was no doubt about whether complete axonal transection was achieved.

While a great deal of attention has been paid to the acute stage of SCI, there have been few studies concerning axonal regeneration in the chronic period of complete paralysis following SCI despite the majority of patients being chronic paralysis patients.

The model developed in the present study was also based on the assumption that glial scar tissue inhibits axonal regeneration in chronic SCI.

## Materials and methods

### Animal care

The present study was undertaken at the Laboratory for Experimental Studies of Yamaguchi University in accordance with the Guidelines for Animal Experiments at Yamaguchi University School of Medicine and the Law and Notification of the Government, following the approval of the experimental design by the Committee on the Ethics of Animal Experiments at Yamaguchi University School of Medicine.

Adult, female Wistar rats (220–250 g) were used in the study. The animals were provided with an ordinary laboratory diet and water. The rats were housed in cages in rooms with controlled lighting and temperature. Female rats were used due to the ease of managing their bladder expression in SCI experiments.

### Surgical method

The surgery was performed under anesthesia by an intramuscular injection of ketamine (60 mg/kg; Sankyo Co. Ltd., Tokyo, Japan) and xylazine (5 mg/kg; Bayer AG, Leverkusen, Germany), with the assistance of a surgical microscope. Rats were positioned on the special operating table in the prone position, immobilized and the hair on the back area was shaved. Following a Th5-12 midline skin incision and paravertebral muscle dissection, the spinous processes and laminar arcs of Th7-9 were removed.

**Figure 1.**
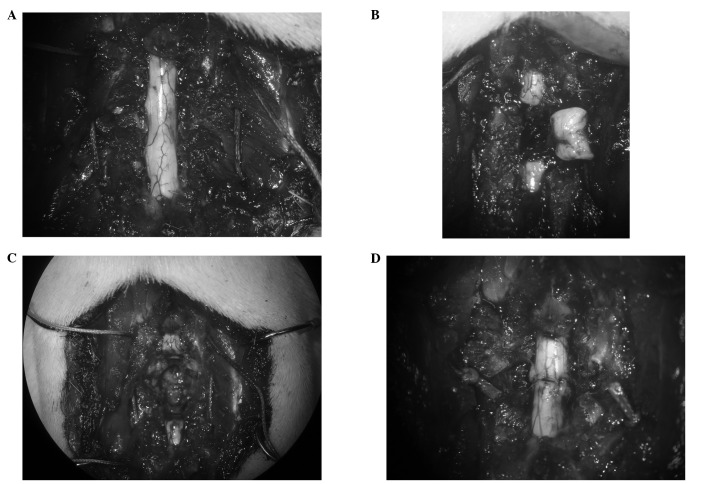
Surgical procedure for shortening the spine and spinal cord. (A) Spinous processes and laminar arcs of Th7-9 were removed. The 7th and 9th ribs were dissected from the pleura enabling them to be passed by stitches for bringing together and wiring the Th7 spine to the Th9 spine. (B) A 4-mm-long segment of the spinal cord was removed at Th8 using the edge of a razor. (C and D) The Th8 vertebra was removed. The stitches which had been passed by the 7th and 9th ribs bilaterally were tied gradually to bring the stumps of the spinal cord together.

The 7th and 9th ribs were dissected from the pleura to allow them to be passed by stitches (3/0 polyamide multi-filament) for bringing together and wiring the Th7 spine to the Th9 spine (Fig. 1A). The spinal cord was exposed and a 4-mm-long segment was removed at Th8 (Fig. 1B) using the edge of a razor (FEATHER Safety Razor Co., Ltd., Osaka, Japan). Care was taken to transect the spinal cord with as sharp a razor as possible to minimize traumatic injury. The 8th rib was freed from the pleura and dissected from the costovertebral joint. Subsequently, the Th7/8 and Th8/9 discs were cut between the stumps of the spinal cord using a sharp-pointed knife to remove the Th8 vertebra (Fig. 1C). The stitches which had been passed by the the 7th and 9th ribs bilaterally were tied gradually to bring together the stumps of the spinal cord (Fig. 1D). Finally, the muscle and the incision were sutured with a 3/0 polyamide multifilament.

Following the surgical procedure, the rats were placed in a warming chamber for a number of days to maintain their body temperature. Manual bladder expression was performed twice a day until the voiding reflexes were reestablished.

### Scaffold model

This model used a scaffold to bridge the defect of the spinal cord. A total of 4,000 collagen filaments, each 20 μm in diameter and made of highly purified type 1 atelocollagen, were used to create the nerve scaffold (5–10). A 9-mm-long segment of the spinal cord was removed, producing a gap of ∼5 mm in the spinal cord following the shortening of the spine. A scaffold of almost the same size as the resected portion was then implanted in the gap.

### Histological analysis

After 12 weeks, the animals were deeply anesthetized and an intracardiac perfusion was performed with isotonic saline for 5 min, followed by 4% paraformaldehyde in 0.1 M phosphate-buffered saline (PBS) for 5 min. Following the perfusion, the thoracic spine and spinal cord were removed.

The spinal cord tissue was immersed in paraformaldehyde in 0.1 M PBS at 4°C overnight. The samples were cut longitudinally into 6-μm thick sections which were stained with hematoxylin and eosin (H&E). The sections were then examined by light microscopy.

## Results

### Transected SCI model

The procedure did not require the aid of an assistant. A few rats operated on initially died in the intraoperative period and the deaths were attributed to pneumothorax caused by the rupture of the pleura during the removal of the Th8 vertebra. Almost all the rats survived until the end of the experiment.

**Figure 2.**
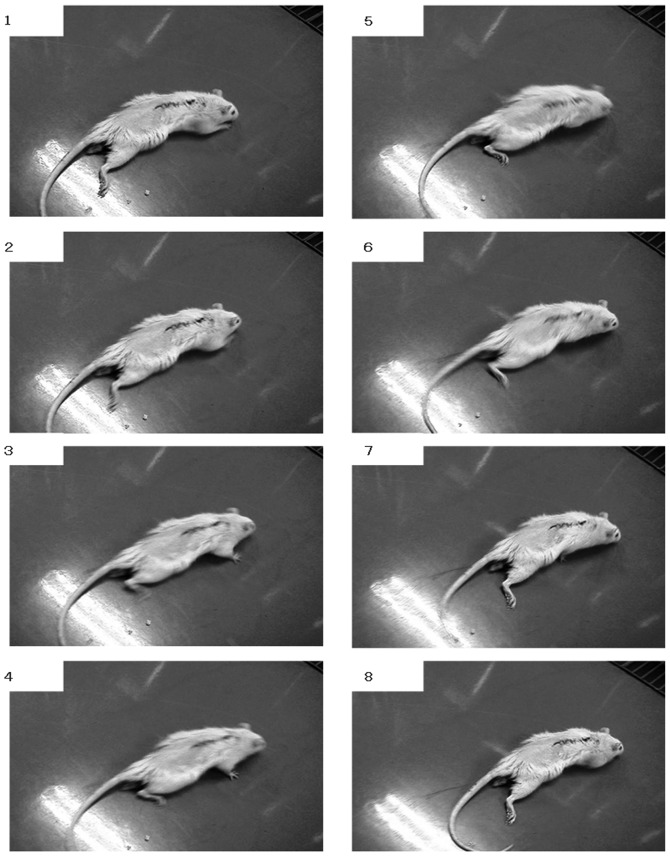
Motor function at 4 weeks after surgery. The sequence of photographs shows the uncoordinated movement of the hind limbs in locomotion at 4 weeks after surgery. However coordinated movements of the hind limbs in locomotion were not observed until the end of the experiment.

The rats had complete flaccid paraplegia immediately after the cord resection. The hind limbs were flaccid in all rats 1 week after the surgery. The rats dragged themselves around with their forelimbs and the hind limbs were stretched out behind. The scratch reflex of the hind limbs was detected at 2–3 weeks after surgery. Uncoordinated movements of the hind limbs in locomotion were observed at 4 weeks after surgery (Fig. 2). However coordinated movements of the hind limbs in locomotion were not observed until the end of the experiment and the maximum Basso-Beattie-Bresnahan (BBB) score was 4.

### Spinal analysis

**Figure 3.**
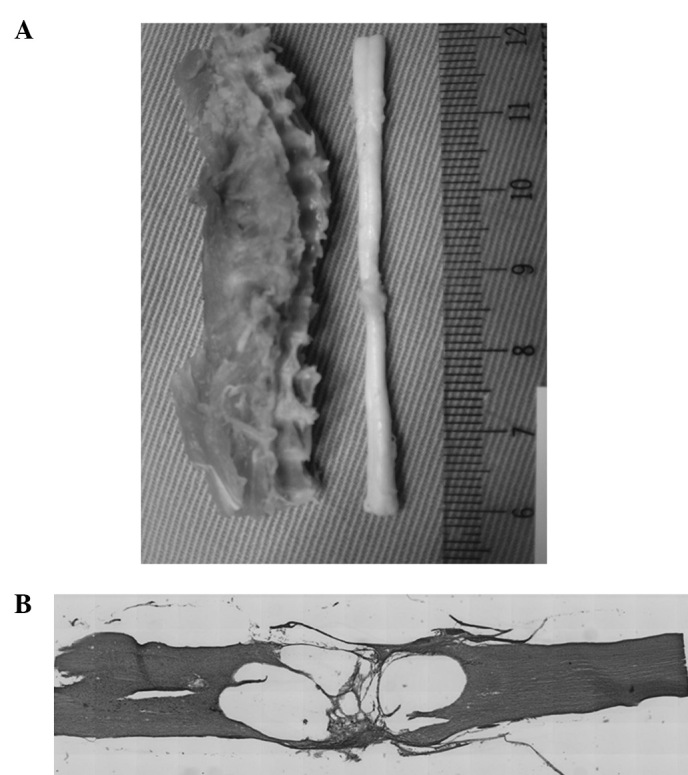
Results of spine and spinal cord shortening. (A) Bone fusion of the Th7 and Th9 vertebrae was observed to be complete and the alignment of thoracic spine was maintained. The spinal canal was also correctly reconstituted. The stumps of the spinal cord were connected. Spinal cords were slightly atrophic around the connection site. (B) Light microscopy of the cord showed that scar tissue intervened at the connection site. Cavitation inhibiting the axonal regeneration was also observed.

No signs of infection were observed in the thoracic spine and spinal cord. The bone fusion of the Th7 and Th9 vertebrae was observed to be complete in all specimens and the alignment of the thoracic spine was maintained. The spinal canal was also correctly reconstituted. Although the dura adhered to the bone at the site of the surgery, the stumps of the spinal cord were connected. The spinal cords were slightly atrophic around the connection site (Fig. 3A).

Light microscopy of the cord showed that scar tissue intervened at the connection site. Cavitation inhibiting the axonal regeneration was also observed (Fig. 3B).

### Scaffold model

**Figure 4.**
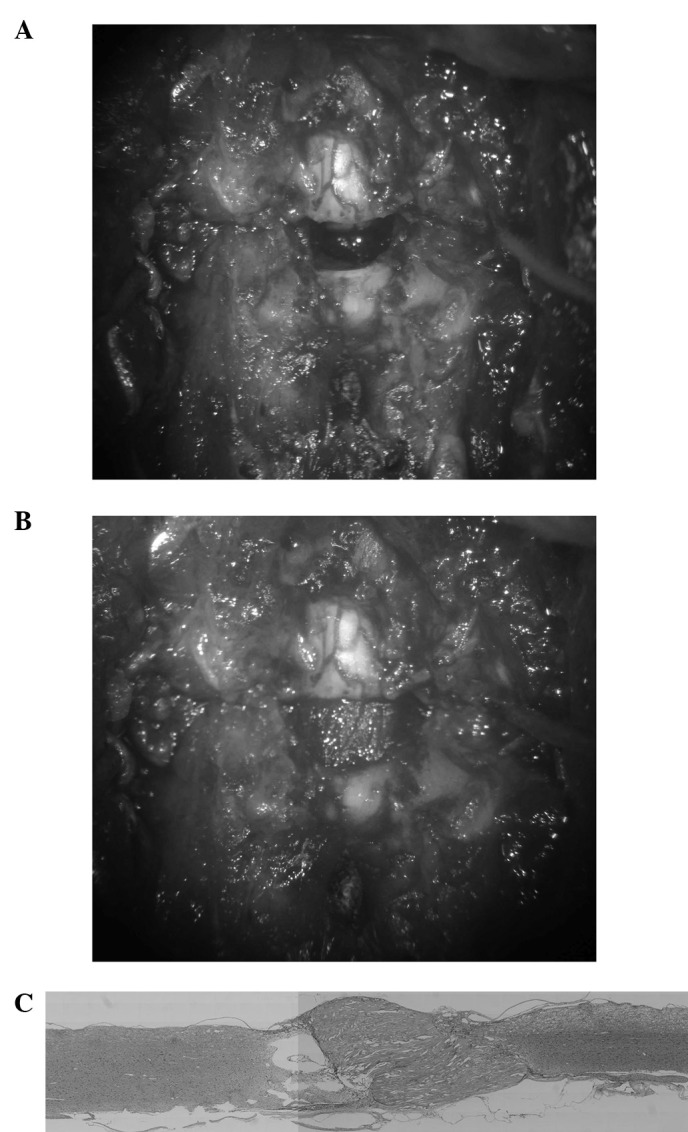
Scaffold model. (A) Photograph showing the model. A gap of ∼5mm in the spinal cord after shortening of the spine. (B) A scaffold of almost the same size as the resected portion was then implanted in the gap. (C) Light microscopy of the cord showed that the implant firmly connected the stumps of the spinal cord and scar tissue and cavitation were less than in previous models.

A 9-mm-long segment of the spinal cord was removed to produce a gap of ∼5 mm after the shortening of the spine (Fig. 4A). A nerve scaffold created from collagen filaments was then implanted in the gap (Fig. 4B). The implant firmly connected the stumps of the spinal cord and the scar tissue and cavitation were less than in previous models (Fig. 4C).

## Discussion

Although axonal regeneration is extremely limited in the mammalian adult central nervous system, partial lesions of the spinal cord may be followed by spontaneous functional improvements. The mechanisms underlying this recovery are not fully understood.

Certain studies have presented evidence that the regeneration of axons in the spinal cord may occur following spinal cord transection in young rats (11–16). However, in a transection-regeneration model, the completeness of the transection has always been a matter of dispute.

Studies have demonstrated that the injured spinal cord spontaneously forms a new intraspinal circuit in adult rats. Transected hind limb corticospinal tract axons have been reported to sprout into the cervical gray matter to contact short and long propriospinal neurons following incomplete SCI (17,18). There is a great difference between complete transection and incomplete transection in the rat model. If the spinal cord had not been transected completely, it is possible that the sprouted axon may have remained following the transection rather than having been regenerated across the transected spinal cord. This could not be resolved by any transected spinal cord model.

We therefore established a method for shortening the rat spine and spinal cord to provide a SCI model in which there was no doubt about whether the axonal transection was complete.

Thoracic spondylectomy in the rat model is difficult due to the adhesion of the pleura and mediastinal organs to the ribs and column (19). Nevertheless the thoracic level was selected for several reasons: i) the spinal cord at the thoracic level is adequate for the evaluation of corticospinal tract axonal regeneration; ii) mechanical stress was observed to be low due to stabilization by the rib so the chance of secondary displacement was lower following thoracic spondylectomy; and iii) the rib is utilized for the shortening and fixation of the spine.

Axonal regeneration was not observed across the transected spinal cord in this model. Scar tissue and cavitation inhibited axonal regeneration at the connection site.

The sharpness of the transection was considered to be one of the most important factors for successful axonal regeneration. An extremely sharp transection produced edema-free lesions and later formed neither cysts nor scars, whereas a relatively blunt transection produced edema followed by scars and cysts around the lesions. Consequently, the spinal cord was transected using the edge of a razor which was as sharp as possible to minimize traumatic injury. However, the stump of the spinal cord resulted in edema since it took 10 or 20 min to bring together the stumps of the spinal cord following tran-section. It is necessary to minimize the damage to the spinal cord and the surgery time for successful axonal regeneration to occur.

Less scar formation and cavitation was observed in the collagen filament model. It is possible that the contact guidance by the collagen filaments guided the regeneration of the axons and resulted in reduced scar formation.

In future, this model may be applied to the chronic period of complete paralysis following SCI. Our model may be useful in the study of axonal regeneration in SCI.

In conclusion, we established a method for shortening the rat spine and spinal cord to provide a SCI model in which there was no doubt about whether the axonal transection was complete. This model was based on the assumption that the glial scar tissue inhibits axonal regeneration in chronic SCI. Axonal regeneration was not observed across the transected spinal cord in this model. For successful axonal regeneration to occur, damage to the spinal cord and the surgery time should be minimized. Our model may be useful in the study of axonal regeneration in SCI.
